# Measuring the magnetic axis alignment during solenoids working

**DOI:** 10.1038/s41598-018-29667-1

**Published:** 2018-07-30

**Authors:** Pasquale Arpaia, Biase Celano, Luca De Vito, Antonio Esposito, Alessandro Parrella, Alessandro Vannozzi

**Affiliations:** 1 Instrumentation and Measurement for Particle Accelerators Lab (IMPALab), Department of Electrical Engineering and Information Technology, University of Naples Federico II, Naples, Italy; 2 National Institute for Nuclear Physics (INFN), Naples, Italy; 3 Department of Engineering, University of Sannio, Benevento, Italy; 4 Instituto Superior Tecnico, University of Lisbon, Lisbon, Portugal; 5 INFN-LNF Istituto Nazionale di Fisica Nucleare Accelerator Division - Magnets and Power Supplies Via E. Fermi, 40 - I-00044 Frascati Rome, Italy; 6 CRdC Tecnologie Scarl, Neaples, Italy

## Abstract

A method for monitoring the misalignment of the magnetic axis in solenoids is proposed. This method requires only a few measurements of the magnetic field at fixed positions inside the magnet aperture, and thus overcomes the main drawback of sturdy moving mechanics of other Hall sensor-based methods. Conversely to state-of-the-art axis determination, the proposed method can be applied also during magnet operations, when the axis region and almost the whole remaining magnet aperture are not accessible. Moreover, only a few measurements of the magnetic field at fixed positions inside the magnet aperture are required: thus a slow process such as the mapping of the whole aperture of a magnet by means of moving stages is not necessary. The mathematical formulation of the method is explained, and a case study on a model of a multi–layer solenoid is presented. For this case study, the uncertainty is assessed and the optimal placement of the Hall transducers is derived.

## Introduction

In the last decades, resistive and superconductive axially-symmetric magnets have been investigated and employed at an increasing rate in several and heterogeneous research fields^1–3^. As an example, in particle accelerators, magnetic elements such as solenoids are applied in low-energy beam transport sections^4,5^, and in modern radio-frequency (RF) linear accelerators (linacs)^6^, for emittance compensation, transverse focusing, and electron cooling. Examples of resistive solenoids employed as focusing lenses in RF linacs are Linac3^7^ and Linac4^8^ at CERN. Superconductive focusing solenoids are adopted also for the project High Intense Neutrino Source (HINS) at Fermilab^9^ and for the High Intensity and Energy (HIE) upgrade of the ISOLDE facility at CERN^10^. The advantage of using short solenoidal lenses in high-power accelerators, instead of sequential pairs of quadrupoles, is the reduction of the emittance growth and the related particle losses^11^. However, the application of axially-symmetric magnets goes beyond the particle accelerator research. For instance, a low-field, large-bore High Temperature Superconductive solenoid for emittance compensation was designed for the superconducting radio frequency electron gun for the WiFEL at the University of Wisconsin^12^. Magnets of this type have also been employed in many different devices, such as electron microscopes^13^, particle therapy, and short-pulse radiographic diagnostics^14^.

An axially-symmetric magnet is based on one or a series of axially-centered coils, producing a region of cylindrically-symmetric, radial, and axial magnetic fields. The solenoid field consists of two components: a dominant, axial component, with a maximum strength at the center of the solenoid, and a weak, radial component with relevant effects only towards the ends of the solenoid. Charged particles moving outside the magnetic axis are azimuthally accelerated by the radial field component, especially in the magnet’s end regions. This leads to helical motion of the charged particles in the longitudinal field region of the magnet. Therefore, particle beams require a strict determination of the magnetic axis. Axially-symmetric magnets are hardly compatible with the standard instrumentation optimized for accelerator multi-pole magnets and are routinely tested with expensive and time-consuming mapping systems^15^. Recently, several methods have been developed to measure the magnetic field of axially-symmetric magnets and overcome the use of mappers. For instance, a novel method exploiting the inherent axial symmetry of the magnetic field was proposed. The method in^3,16^ is based on an induction transducer, sensitive to the longitudinal and radial components of the solenoid under test, moving along the magnet axis. The voltage induced in the transducer is then acquired and integrated digitally in order to yield the flux linkage as a function of the linear position, measured by a laser interferometer. In the literature, the methods to align solenoids can be grouped in three main categories^17^: (i) the single stretched-wire methods^18,19^, (ii) the vibrating-wire methods^20–22^ and (iii) the Hall transducer-based methods^15,23^.

The single-stretched-wire method exploits the Faraday induction law: when a single conducting wire is moved inside a magnetic field, the integrated voltage across its connection terminals is a measurement of the magnetic flux linked with the surface traced out by the wire. The axis is obtained by iterating horizontal and vertical sweeps of the wire until symmetric start and end points, where the flux is null, are found. This is a standard method for finding quadrupole magnets’ magnetic axes; however, in the case of solenoids, this method has a much lower sensitivity, because the intercepted transversal field components are significant only at the magnet’s ends. The vibrating-wire method, instead, is based on the Lorentz force. When a current pulse is driven through the wire, its interaction with the magnetic field generates mechanical vibrations, which can be measured and put in relation with the surrounding field. This method is a standard to measure magnetic axis position in quadrupole magnets for particle accelerators. The basic idea is to find the wire position in the magnet aperture where the smallest oscillations at first and second resonance frequencies are observed^21^. This same principle was applied to solenoids^20^. When the wire position coincides with the magnetic axis, the transversal field components cancel out and no motion is induced on the wire. Two wire resonance frequencies are excited for co-and counter-directional movements of the wire stages in the process of centering and aligning a solenoid. This procedure of finding the minimum oscillation amplitudes has a sensitivity to the misalignment in the order of the micrometer. The main drawback of this method is that the procedure is applicable only if the whole solenoid’s aperture is accessible. Hence, these methods cannot be applied to real-time monitoring of an operational solenoid.

A different approach for estimating the magnetic axis consists of using Hall transducers. For example, the field generated by the rotation of a Hall transducer at several points along the axis of a solenoid can be recorded as a function of the rotation angle^23^. Then, the displacement of the axis of rotation from the magnetic axis is retrieved post-processing the solenoid field. A common way to determine the magnetic axis by means of Hall transducers to calculate the magnetic center is by measuring the 2D field profile of the solenoid at different positions along the z-axis and then estimating the magnetic axis position with a resolution of 0.01 mm^15^. In this case, the axial component of the magnetic field is measured, rather than the radial component. The drawback of the Hall transducer-based methods is the mapping of the whole solenoid aperture, generally performed with moving stages through sturdy mechanics. When a magnet is in operation, its coil is constantly subjected to an electrodynamic strain. This could easily bring to a significant misalignment of the magnetic axis from the geometric axis. In case of a multi-coil solenoid this effect would be even more dramatic, because the different coils could show different misalignments. Hence, a system for monitoring in real time these misalignments is essential in all applications where a strict constraint on the coils alignment is required, giving the possibility to adjust each coil position to achieve/recover the solenoid design parameters. However, the standard methods to find the magnetic axis, introduced in this section, are not suitable for a real-time monitoring, because they require that the whole solenoid’s aperture is accessible.

In this paper, a novel Hall sensors-based method is proposed for the real-time monitoring of solenoids magnetic axis misalignment when the aperture is not accessible. Only a few measurements of the magnetic field at fixed positions inside the magnet aperture are required: thus a slow process such as the mapping of the whole magnet aperture by means of moving stages is not necessary. With respect to the preliminary version^24^ based on tri-axial Hall transducers, less expensive and more accurate uni-axial transducers are exploited. Moreover, a study on the uncertainty of the method is presented. In particular, Section 2 presents the mathematical description of the method, discussing the involved geometrical quantities and two different formulations for four and three transducers per side. In Section 3, a case study on a model of a multi–layer solenoid is illustrated, by assessing also the measurement uncertainty and optimizing the placement of the Hall transducers.

## Proposed Method

### Basic idea

**Figure 1:**
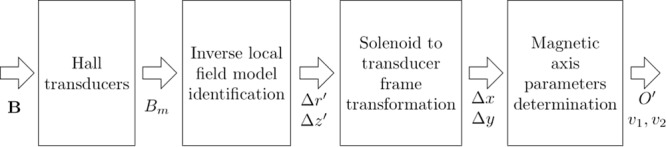
Main steps of the measurement method.

The proposed method aims at measuring the misalignment of the magnetic axis of a solenoid by using the difference between measurements of magnetic flux density in the actual and aligned cases. The method is summed up in Fig. 1. The magnetic flux density is measured by two sets of Hall transducers, placed on two planes orthogonal to the nominal axis at the extremities of the solenoid.

The measured values *B*_*m*_(*H*_*hk*_) at each position *H*_*hk*_ of the transducers are exploited to identify an inverse local field model, defined by linearizing the magnetic flux density around the positions where the transducers would move, due to the axis misalignment. The model allows to compute the displacements Δ*z*′ and Δ*r*′ of the transducer coordinates in the solenoid frame from the combination of the magnetic flux densities measured by the transducers. The displacements Δ*z*′ and Δ*r*′ are then transformed to the transducer frame and related to the Cartesian displacements Δ*x*, Δ*y* of the magnetic axis interception with the transducers planes. Finally, these displacements are used to get the parametric equation of the magnetic axis.

### Analytical statement of the method

The proposed method assumes that:

- From the solenoid point of view, the magnetic flux density preserves its axisymmetry even when the coil is misaligned with respect to the desired position.
- The displacements of the interceptions of the magnetic axis with the planes of the transducers are small with respect to the radial position of the transducers. This assumption will be better clarified within the discussion.
- The Hall transducers are not subject to movements.
- There is no longitudinal shift for the solenoid.

For an axially-symmetric magnet, the magnetic flux density **B** expressed in the cylindrical coordinate system (*r*, *φ*, *z*) is independent from the angle *φ*. This means that the components of the field, namely *B*_*r*_, *B*_*z*_, and *B*_*φ*_, are all independent of *φ*. Furthermore, for a solenoid, it is also *B*_*φ*_ = 0, because **B** is obtained as the curl of a vector potential **A** = ${A}_{\phi }\hat{\phi }$, which has only a component along $\hat{\phi }$. Due to the axisymmetry, considering a circle with radius *R*_0_ centered on the solenoid axis and contained in a plane orthogonal to this same axis, the two components of **B** will have a constant magnitude for all the points of the circle. This implies that also the field module B is constant on such a circle.

**Figure 2:**
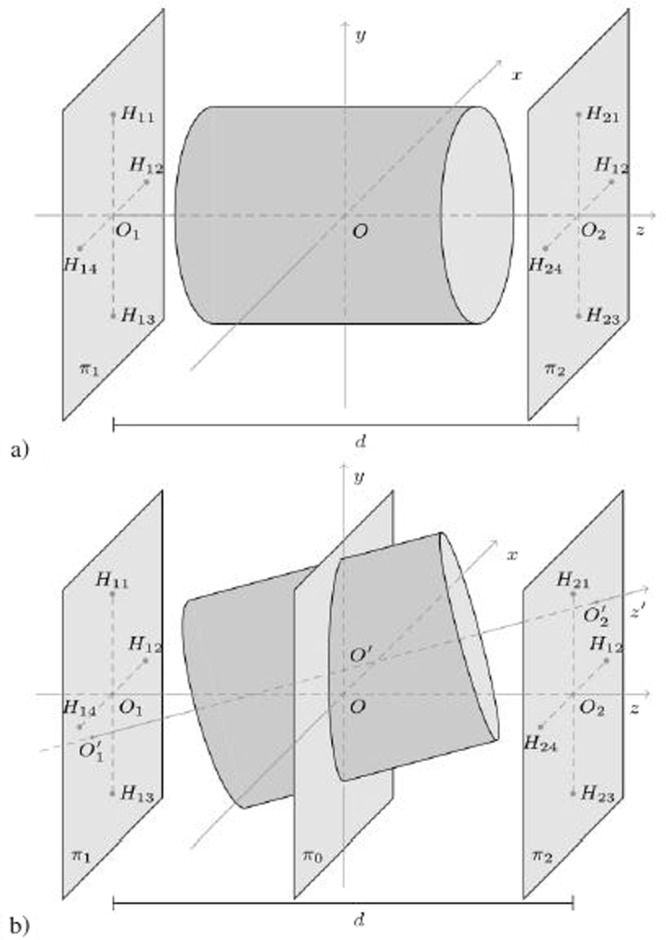
(**a**) Aligned solenoid: the magnetic axis matches with the *z* axis of the transducers reference system (reference axis). (**b**) Misaligned solenoid: the magnetic axis (*z*’) differs from the reference axis (*z*), but it can be identified through ${O}_{1}^{^{\prime} }$ and ${O}_{2}^{^{\prime} }$.

When the solenoid is in its nominal position (*aligned solenoid*, Fig. 2a), a Cartesian reference system with the origin at the center of the solenoid (*O*), and the *z* axis along the solenoid magnetic axis can be employed. Let us assume that eight Hall transducers are placed at the coordinates (−*R*_0_, 0), (+*R*_0_, 0), (0, −*R*_0_), (0, +*R*_0_) of the planes *π*_1_ and *π*_2_. Such planes are orthogonal to the *z* axis, and at positions *z* = ±*d*/2, with *d* the distance between them. The solenoid magnetic axis passes through the points *O*_1_ and *O*_2_, representing the intersection of the planes *π*_1_ and *π*_2_ with the *z* axis. Meanwhile, the Hall transducers will be subjected to magnetic flux densities with same amplitude, but with different orientation, because they are placed at the same distance from the magnetic axis.

When the solenoid is out from its nominal position (*misaligned solenoid*, Fig. 2b), the magnetic flux densities **B** measured by the transducers are different not only in the orientation but also in the magnitude, owing to the lack of symmetry (the transducers are assumed as not subject to movements). The above mentioned solenoid reference system is defined as a coordinate system coinciding with the transducers reference when the solenoid is aligned.

In the solenoid reference (Fig. 2b), the coordinates are pointed out with an apostrophe (′), the origin is the center of the misaligned solenoid (*O*′), and the magnetic axis corresponds to the *z*’ axis. The magnetic axis is uniquely determined by the coordinates of the solenoid center *O*′ ≡ (*x*_*O*′_, *y*_*O*′_, *z*_*O*′_), and by its slope parameters *v*_1_, *v*_2_, *v*_3_:

1 $$\{\begin{array}{l}x={x}_{O^{\prime} }+{v}_{1}t\\ y={y}_{O^{\prime} }+{v}_{2}t\\ z={z}_{O^{\prime} }+{v}_{3}t\end{array}$$

Indicating with ${O^{\prime} }_{1}$ and ${O^{\prime} }_{2}$ the intersections of the magnetic axis with the planes *π*_1_ and *π*_2_, respectively, the slope parameters are obtained as:

2 $$\begin{array}{l}{v}_{1}={x}_{{O}_{2}^{^{\prime} }}-{x}_{{O}_{1}^{^{\prime} }}\\ {v}_{2}={y}_{{O}_{2}^{^{\prime} }}-{y}_{{O}_{1}^{^{\prime} }}\\ {v}_{3}={z}_{{O}_{2}^{^{\prime} }}-{z}_{{O}_{1}^{^{\prime} }}\end{array}$$

Then, according to the assumption of lack of longitudinal shift, the solenoid center is constrained to the plane *π*_*o*_: *z* = 0 (Fig. 2b). Therefore, only radial displacements and tilt are considered; thus, the solenoid does not move along the *z*-axis. In this case, eqs (1) and (2) are simplified as:

3 $$\{\begin{array}{ll}\begin{array}{l}x={x}_{O^{\prime} }+{v}_{1}t\\ y={y}_{O^{\prime} }+{v}_{2}t\\ z=d\cdot t\end{array} & \begin{array}{l}{v}_{1}={x}_{{O}_{2}^{^{\prime} }}-{x}_{{O}_{1}^{^{\prime} }}\\ {v}_{2}={y}_{{O}_{2}^{^{\prime} }}-{y}_{{O}_{1}^{^{\prime} }}\end{array}\end{array}$$

### Local field model

According to the above assumption of small solenoid axis misalignment, the distance between *O*_1_ ≡ (0, 0, −*d*/2) and ${O^{\prime} }_{1}$ and the distance between *O*_2_ ≡ (0, 0, *d*/2) and ${O^{\prime} }_{2}$ are small with respect to *R*_0_:

4 $$\begin{array}{rcl}|{O^{\prime} }_{1}-{O}_{1}| & = & \sqrt{{x}_{{O^{\prime} }_{1}}^{2}+{y}_{{O^{\prime} }_{1}}^{2}}\ll {R}_{0}\\ |{O^{\prime} }_{2}-{O}_{2}| & = & \sqrt{{x}_{{O^{\prime} }_{2}}^{2}+{y}_{{O^{\prime} }_{2}}^{2}}\ll {R}_{0}\end{array}$$

Under this assumption, the field flux density is approximated with its first-order Taylor expansion around the position ${H^{\prime} }_{hk}$, corresponding to the point where the transducer would move, owing to the misalignment. Then, using such an expansion, the magnetic flux density is assessed in *H*_*hk*_ (the position where the transducer actually lies). Here the subscript “h” identifies the plane where the transducer is located (*h* = 1 for *π*_1_, *h* = 2 for *π*_2_), while “k” identifies the exact transducer of the plane.

**Figure 3:**
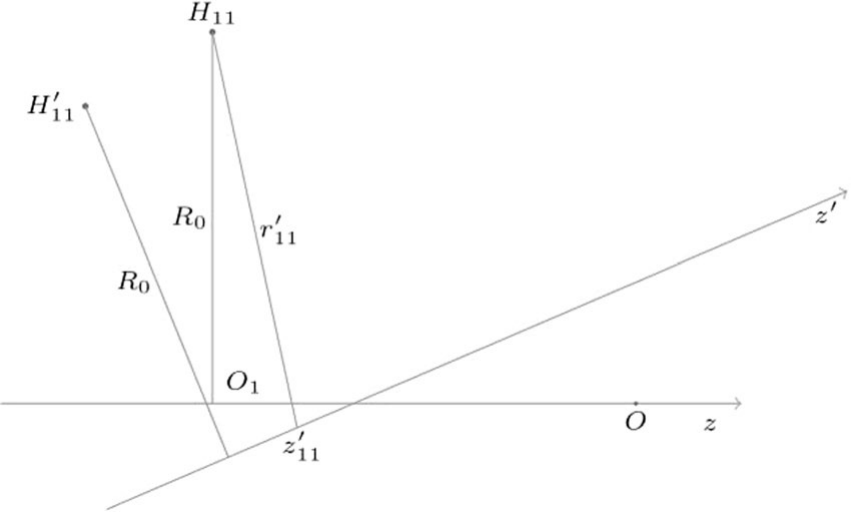
The position of the transducer *H*_11_ in both the transducer and the solenoid reference systems. The magnetic flux density is expanded in Taylor series around the position ${H^{\prime} }_{11}$, corresponding to the point where *H*_11_ would move due to the misalignment.

The geometry suggests to adopt cylindrical coordinates, and, in the solenoid reference system, the magnetic flux density only depends on the radial (*r*′) and axial (*z*′) coordinates, but not upon the angle (*φ*′), owing to the axisymmetry. As an example, the case of one of the transducers is highlighted in Fig. 3. The transducer lies on the *H*_11_ position, moving to ${H^{\prime} }_{11}$ owing to the misalignment. In this case, the expansion will be around the point ${H^{\prime} }_{11}$, having coordinates *r*′ = *R*_0_, *z*′ = −*d*/2 in the solenoid reference system.

In the case of a generic transducer, located at *H*_*hk*_, the magnetic flux density is expanded as:

5 $${\bf{B}}({r^{\prime} }_{hk},{z^{\prime} }_{hk})\approx {\bf{B}}({R}_{0},{z}_{h})+{\frac{\partial {\bf{B}}}{\partial r}|}_{{R}_{0},{z}_{h}}({r^{\prime} }_{hk}-{R}_{0})+{\frac{\partial {\bf{B}}}{\partial z}|}_{{R}_{0},{z}_{h}}({z^{\prime} }_{hk}-{z}_{h}),$$

where, *z*_*h*_ = ±*d*/2, depending on the side of the solenoid where the transducer is placed. The first member of the equation is the quantity to be measured. In particular, uni-axial Hall transducers can be adopted, positioned along either the radial or the longitudinal direction of the transducer reference system. 1D Hall transducers placed radially are first considered.

**Figure 4:**
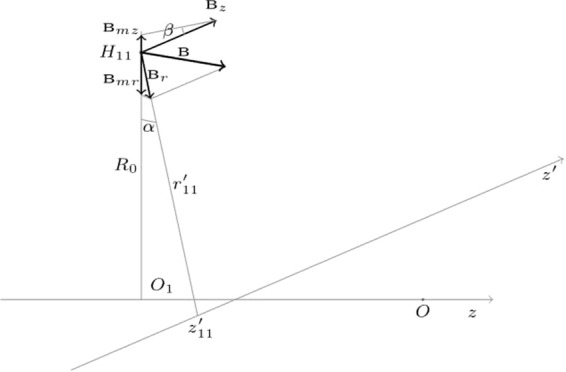
**B**_*r*_ and **B**_*z*_ orientation with respect to the radial direction on which the considered transducer is placed.

A radial transducer measures only the radial component of **B** when the solenoid is aligned. Instead, when the solenoid is misaligned, also a contribution from the axial component of the field will appear. The measured value is then composed by the superposition of the projections of **B**_***r***_ and **B**_*z*_ onto the radial direction of the transducer reference system. It will then depend on the magnitudes *B*_*r*_, *B*_*z*_, and on the angles *α* and *β*, between the vectors **B**_*r*_ and **B**_*z*_ and the radial direction of the transducer reference system, respectively (Fig. 4), again for a single transducer. The measured value is then expressed by:

6 $${B}_{m}={B}_{mr}+{B}_{mz}={B}_{r}\,\cos \,\alpha +{B}_{z}\,\sin \,\beta $$

where the projections assume negative values when directed towards the *z*-axis and vice versa. As an example, in the case of Fig. 4, *B*_*mz*_ has a positive value, while *B*_*mr*_ a negative value.

Under the assumption of small misalignment, eq. (6) can be simplified by assuming cos *α* ≈ 1. Then, the sign of *B*_*mr*_ and *B*_*r*_ coincide. Instead, the sign of *B*_*mz*_ coincides with the sign of sin *β* because *B*_*z*_ is always positive. Here, *B*_*r*_ and *B*_*z*_ can be expressed by Taylor expansion, as shown in (5). Such an expansion depends on the coordinates $({r^{\prime} }_{hk},{z^{\prime} }_{hk})$ of the transducer positions in the solenoid reference system. The derivation of the geometrical quantities as well as of sin *β* for the different transducers is reported in the next subsection.

### Geometrical quantities

**Figure 5:**
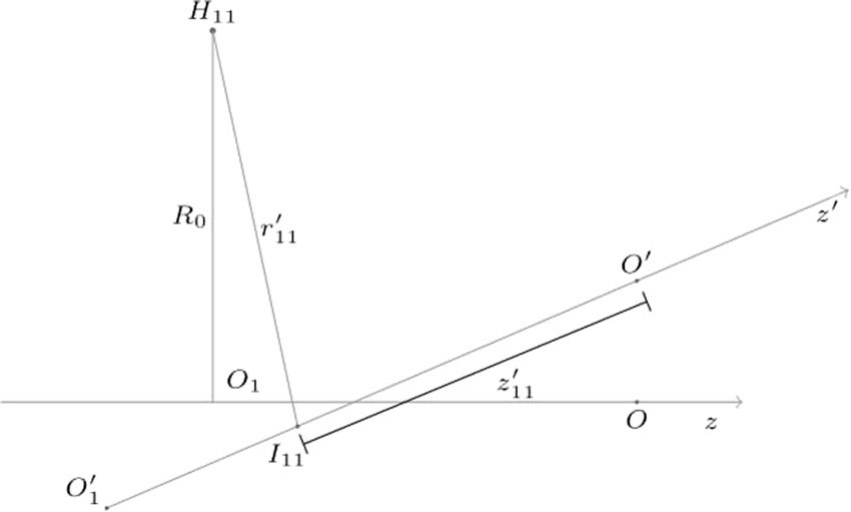
Coordinates $({r^{\prime} }_{11},{z^{\prime} }_{11})$ of the transducer position in the solenoid reference system.

The coordinates $({r^{\prime} }_{hk},{z^{\prime} }_{hk})$ of *H*_*hk*_ in the solenoid reference system (Fig. 5) can be expressed in terms of the points *H*_*hk*_, *I*_*hk*_, and ${O^{\prime} }_{1}$ (or ${O^{\prime} }_{2}$), in the transducers reference. *H*_*hk*_ is the point where a transducer is located, *I*_*hk*_ its projection on the magnetic axis, and ${O^{\prime} }_{1}$ the intersection between the magnetic axis and the plane *π*_1_ considered in the figure.

${r^{\prime} }_{hk}$ is the distance of the transducer position from the solenoid axis, namely the distance between *H*_*hk*_ and ${I}_{hk}\equiv ({x}_{{I}_{hk}},{y}_{{I}_{hk}},{z}_{{I}_{hk}})$. For example, considering *H*_11_ ≡ (0, +*R*_0_, −*d*/2), eq. (7) holds under the hypothesis of small axis misalignment:

7 $$\begin{array}{rcl}{r^{\prime} }_{11} & = & \sqrt{{({x}_{{I}_{11}})}^{2}+{({R}_{0}-{y}_{{I}_{11}})}^{2}+{(-\frac{d}{2}-{z}_{{I}_{11}})}^{2}}\\  & = & {R}_{0}\sqrt{{(\frac{{x}_{{I}_{11}}}{{R}_{0}})}^{2}+{(\frac{{R}_{0}-{y}_{{I}_{11}}}{{R}_{0}})}^{2}+{(\frac{-d/2-{z}_{{I}_{11}}}{{R}_{0}})}^{2}}\\  & \approx  & {R}_{0}\sqrt{{(\frac{{R}_{0}-{y}_{{I}_{11}}}{{R}_{0}})}^{2}}={R}_{0}-{y}_{{I}_{11}}\end{array}$$

Analogously, in the case of *H*_13_ ≡ (0, −*R*_0_, −*d*/2):

8 $${r}_{13}^{^{\prime} }\approx {R}_{0}\sqrt{{(\frac{-{R}_{0}-{y}_{{I}_{13}}}{{R}_{0}})}^{2}}={R}_{0}+{y}_{{I}_{13}}$$

The points *I*_*hk*_ can be described with the parametrization of the magnetic axis introduced in eq. (3). The equation of the *z*′ axis must be combined with the equation describing a plane orthogonal to *z*′ and passing through the transducer ${H}_{hk}\equiv ({x}_{{H}_{hk}},{y}_{{H}_{hk}},{z}_{{H}_{hk}})$, namely the expression (9).

9 $${v}_{1}(x-{x}_{{H}_{hk}})+{v}_{2}(y-{y}_{{H}_{hk}})+d(z-{z}_{{H}_{hk}})=0$$

It results that *I*_*hk*_ has the following parametric expression:

10 $$\{\begin{array}{rcl}{x}_{{I}_{hk}} & = & {x}_{O^{\prime} }+{t}_{{I}_{hk}}{v}_{1}\\ {y}_{{I}_{hk}} & = & {y}_{O^{\prime} }+{t}_{{I}_{hk}}{v}_{2}\\ {z}_{{I}_{hk}} & = & {t}_{{I}_{hk}}d\\ {t}_{{I}_{hk}} & = & \tfrac{{v}_{1}({x}_{{H}_{hk}}-{x}_{O^{\prime} })+{v}_{2}({y}_{{H}_{hk}}-{y}_{O^{\prime} })+d{z}_{{H}_{hk}}}{{v}_{1}^{2}+{v}_{2}^{2}+{d}^{2}}\approx \tfrac{{v}_{1}({x}_{{H}_{hk}}-{x}_{O^{\prime} })+{v}_{2}({y}_{{H}_{hk}}-{y}_{O^{\prime} })+d{z}_{{H}_{hk}}}{{d}^{2}}\end{array}$$

${z^{\prime} }_{hk}$ is instead the distance of *I*_*hk*_ from the solenoid center. Actually, in the Taylor expansion, the term ${z^{\prime} }_{hk}-{z}_{h}$ is needed, and this can be obtained from the third equation of (10), by considering that for *z*_1_ and *z*_2_, is *t* = −1/2 and *t* = 1/2, respectively. Therefore, the following equations apply:

11 $$\begin{array}{rcl}{z^{\prime} }_{1k}-{z}_{1} & = & ({t}_{{I}_{1k}}+\frac{1}{2})d\\ {z^{\prime} }_{2k}-{z}_{2} & = & ({t}_{{I}_{2k}}-\frac{1}{2})d\end{array}$$

In conclusion, the expressions of *B*_*r*_ and *B*_*z*_ can be written with the Taylor expansion thanks to the previous geometrical considerations. As an example, for the transducers in positions *H*_11_ and *H*_13_ it results that:

12 $$\begin{array}{rcl}{B}_{r}({H}_{11}) & \approx  & {B}_{r}({R}_{0},-\,\frac{d}{2})-{\frac{\partial {B}_{r}}{\partial r}|}_{{R}_{0},-\frac{d}{2}}({y}_{O^{\prime} }+{t}_{{I}_{11}}{v}_{2})+{\frac{\partial {B}_{r}}{\partial z}|}_{{R}_{0},-\frac{d}{2}}({t}_{{I}_{11}}+\frac{1}{2})d\\ {B}_{r}({H}_{13}) & \approx  & {B}_{r}({R}_{0},-\,\frac{d}{2})+{\frac{\partial {B}_{r}}{\partial r}|}_{{R}_{0},-\frac{d}{2}}({y}_{O^{\prime} }+{t}_{{I}_{13}}{v}_{2})+{\frac{\partial {B}_{r}}{\partial z}|}_{{R}_{0},-\frac{d}{2}}({t}_{{I}_{13}}+\frac{1}{2})d\end{array}$$

where:

13 $$\begin{array}{rcl}{t}_{{I}_{11}} & = & \frac{{v}_{1}(\,-\,{x}_{O^{\prime} })+{v}_{2}(\,+\,{R}_{0}-{y}_{O^{\prime} })+d(\,-\,d/2)}{{d}^{2}}\approx \frac{{v}_{2}(\,+\,{R}_{0}-{y}_{O^{\prime} })}{{d}^{2}}-\frac{1}{2}\\ {t}_{{I}_{13}} & = & \frac{{v}_{1}(\,-\,{x}_{O^{\prime} })+{v}_{2}(\,-\,{R}_{0}-{y}_{O^{\prime} })+d(\,-\,d/2)}{{d}^{2}}\approx \frac{{v}_{2}(\,-\,{R}_{0}-{y}_{O^{\prime} })}{{d}^{2}}-\frac{1}{2}\end{array}$$

The last term to be determined is sin *β*. For transducers lying on a straight line parallel to the *y*-axis, such as *H*_11_ and *H*_13_, *β* is the elevation angle of the *z*′-axis from the *xz* plane:

14 $$\begin{array}{l}\sin \,{\beta }_{11}=\frac{{y}_{{O}_{2}^{^{\prime} }}-{y}_{{O}_{1}^{^{\prime} }}}{{z}_{{O}_{2}^{^{\prime} }}-{z}_{{O}_{1}^{^{\prime} }}}\approx +\,\frac{{v}_{2}}{d}\\ \sin \,{\beta }_{13}=-\,\sin \,{\beta }_{11}\approx -\,\frac{{v}_{2}}{d}\end{array}$$

Similarly, for transducers lying on a straight line parallel to *x*-axis, such as *H*_12_ and *H*_14_, *β*_12_ is the azimuth angle *z*′-axis from the *yz* plane, and *β*_14_ = −*β*_12_. In the latter case, the following equations apply:

15 $$\begin{array}{rcl}\sin \,{\beta }_{12} & = & \frac{{x}_{{O}_{2}^{^{\prime} }}-{x}_{{O}_{1}^{^{\prime} }}}{{z}_{{O}_{2}^{^{\prime} }}-{z}_{{O}_{1}^{^{\prime} }}}\approx +\,\frac{{v}_{1}}{d}\\ \sin \,{\beta }_{14} & = & -\sin \,{\beta }_{12}\approx -\,\frac{{v}_{1}}{d}\end{array}$$

### Four transducers per side

Let’s consider the case of four uniaxial Hall transducers per solenoid side, placed in the following points:

$$\begin{array}{ll}{H}_{11}\equiv (0,+\,{R}_{0},-\,d/2) & {H}_{21}\equiv (0,+\,{R}_{0},+\,d/2)\\ {H}_{12}\equiv (\,+\,{R}_{0},0,-\,d/2) & {H}_{22}\equiv (\,+\,{R}_{0},0,+\,d/2)\\ {H}_{13}\equiv (0,-\,{R}_{0},-\,d/2) & {H}_{23}\equiv (0,-\,{R}_{0},+\,d/2)\\ {H}_{14}\equiv (\,-\,{R}_{0},0,-\,d/2) & {H}_{24}\equiv (\,-\,{R}_{0},0,+\,d/2)\end{array}$$

In such positions, some symmetries of the magnetic field can be exploited. In the aligned case, indeed, both the radial and the axial components of the magnetic flux density have the same magnitude in the eight considered positions. In particular, the following relations apply:

16 $$\begin{array}{ll}{B}_{r}({H}_{2k})=-\,{B}_{r}({H}_{1k})={B}_{r0} & {B}_{z}({H}_{2k})=+\,{B}_{z}({H}_{1k})={B}_{z0}\\ {\frac{\partial {B}_{r}}{\partial r}|}_{{R}_{0},+\frac{d}{2}}=-\,{\frac{\partial {B}_{r}}{\partial r}|}_{{R}_{0},-\frac{d}{2}}={\frac{\partial {B}_{r}}{\partial r}|}_{0} & {\frac{\partial {B}_{z}}{\partial r}|}_{{R}_{0},+\frac{d}{2}}=+\,{\frac{\partial {B}_{z}}{\partial r}|}_{{R}_{0},-\frac{d}{2}}={\frac{\partial {B}_{z}}{\partial r}|}_{0}\\ {\frac{\partial {B}_{r}}{\partial z}|}_{{R}_{0},+\frac{d}{2}}=+\,{\frac{\partial {B}_{r}}{\partial z}|}_{{R}_{0},-\frac{d}{2}}={\frac{\partial {B}_{r}}{\partial z}|}_{0} & {\frac{\partial {B}_{z}}{\partial z}|}_{{R}_{0},+\frac{d}{2}}=-\,{\frac{\partial {B}_{z}}{\partial z}|}_{{R}_{0},-\frac{d}{2}}={\frac{\partial {B}_{z}}{\partial z}|}_{0}\end{array}$$

The magnetic axis misalignment is sensed by taking into account the difference in the measurements of transducers lying on the same plane and on the same axis (*x* or *y*). As an example, the equation obtained by subtracting the values measured by the couple (*H*_13_, *H*_11_) is shown. Similar equations are then valid for the couples (*H*_14_, *H*_12_), (*H*_23_, *H*_21_), and (*H*_24_, *H*_22_).

17 $$\begin{array}{rcl}{B}_{m}({H}_{13})-{B}_{m}({H}_{11}) & \approx  & [{B}_{r}({H}_{13})-{B}_{r}({H}_{11})]-\frac{{v}_{2}}{d}[{B}_{z}({H}_{13})+{B}_{z}({H}_{11})]\\  & = & -{\frac{\partial {B}_{r}}{\partial r}|}_{0}[2{y}_{O^{\prime} }+({t}_{{I}_{13}}+{t}_{{I}_{11}}){v}_{2}]+{\frac{\partial {B}_{r}}{\partial z}|}_{0}({t}_{{I}_{13}}-{t}_{{I}_{11}})d\\  &  & -\,\frac{{v}_{2}}{d}[2{B}_{z0}+{\frac{\partial {B}_{z}}{\partial r}|}_{0}({t}_{{I}_{13}}-{t}_{{I}_{11}}){v}_{2}-{\frac{\partial {B}_{z}}{\partial z}|}_{0}({t}_{{I}_{13}}+{t}_{{I}_{11}}+1)d]\end{array}$$

Substituting (13) in (17) and neglecting the quadratic terms of *v*_2_:

18 $$\begin{array}{rcl}{B}_{m}({H}_{13})-{B}_{m}({H}_{11}) & \approx  & -{\frac{\partial {B}_{r}}{\partial r}|}_{0}[2{y}_{O^{\prime} }(1-\frac{{v}_{2}^{2}}{{d}^{2}})-{v}_{2}]-2{\frac{\partial {B}_{r}}{\partial z}|}_{0}\frac{{v}_{2}}{d}{R}_{0}\\  &  & -\,\frac{{v}_{2}}{d}[2{B}_{z0}-2{\frac{\partial {B}_{z}}{\partial r}|}_{0}\frac{{v}_{2}^{2}}{{d}^{2}}{R}_{0}-2{\frac{\partial {B}_{z}}{\partial z}|}_{0}\frac{{v}_{2}}{d}{y}_{O^{\prime} }]\\  & \approx  & -2{\frac{\partial {B}_{r}}{\partial r}|}_{0}({y}_{O^{\prime} }-\frac{{v}_{2}}{2})-2\frac{{R}_{0}}{d}{\frac{\partial {B}_{r}}{\partial z}|}_{0}{v}_{2}-2\frac{{v}_{2}}{d}{B}_{z0}\end{array}$$

Repeating the same reasoning for the other transducers couples, the following system of equations is obtained:

19 $$\begin{array}{l}{B}_{m}({H}_{13})-{B}_{m}({H}_{11})=-\,2{\frac{\partial {B}_{r}}{\partial r}|}_{0}({y}_{O^{\prime} }-\frac{{v}_{2}}{2})-2\frac{{R}_{0}}{d}{\frac{\partial {B}_{r}}{\partial z}|}_{0}{v}_{2}-2\frac{{v}_{2}}{d}{B}_{z0}\\ {B}_{m}({H}_{14})-{B}_{m}({H}_{12})=-\,2{\frac{\partial {B}_{r}}{\partial r}|}_{0}({x}_{O^{\prime} }-\frac{{v}_{1}}{2})-2\frac{{R}_{0}}{d}{\frac{\partial {B}_{r}}{\partial z}|}_{0}{v}_{1}-2\frac{{v}_{1}}{d}{B}_{z0}\\ {B}_{m}({H}_{23})-{B}_{m}({H}_{21})=+\,2{\frac{\partial {B}_{r}}{\partial r}|}_{0}({y}_{O^{\prime} }+\frac{{v}_{2}}{2})-2\frac{{R}_{0}}{d}{\frac{\partial {B}_{r}}{\partial z}|}_{0}{v}_{2}-2\frac{{v}_{2}}{d}{B}_{z0}\\ {B}_{m}({H}_{24})-{B}_{m}({H}_{22})=+\,2{\frac{\partial {B}_{r}}{\partial r}|}_{0}({x}_{O^{\prime} }+\frac{{v}_{1}}{2})-2\frac{{R}_{0}}{d}{\frac{\partial {B}_{r}}{\partial z}|}_{0}{v}_{1}-2\frac{{v}_{1}}{d}{B}_{z0}\end{array}$$

This linear system can be easily solved to obtain *x*_*O*′_, *y*_*O*′_, *v*_1_, and *v*_2_:

20 $$\begin{array}{rcl}{x}_{o^{\prime} } & = & \frac{[{B}_{m}({H}_{24})-{B}_{m}({H}_{22})]-[{B}_{m}({H}_{14})-{B}_{m}({H}_{12})]}{4{\frac{\partial {B}_{r}}{\partial r}|}_{0}}\\ {y}_{o^{\prime} } & = & \frac{[{B}_{m}({H}_{23})-{B}_{m}({H}_{21})]-[{B}_{m}({H}_{13})-{B}_{m}({H}_{11})]}{4{\frac{\partial {B}_{r}}{\partial r}|}_{0}}\\ {v}_{1} & = & \frac{[{B}_{m}({H}_{24})-{B}_{m}({H}_{22})]+[{B}_{m}({H}_{14})-{B}_{m}({H}_{12})]}{2({\frac{\partial {B}_{r}}{\partial r}|}_{0}-2\frac{{R}_{0}}{d}{\frac{\partial {B}_{r}}{\partial z}|}_{0}-2\frac{{B}_{z0}}{d})}\\ {v}_{2} & = & \frac{[{B}_{m}({H}_{23})-{B}_{m}({H}_{21})]+[{B}_{m}({H}_{13})-{B}_{m}({H}_{11})]}{2({\frac{\partial {B}_{r}}{\partial r}|}_{0}-2\frac{{R}_{0}}{d}{\frac{\partial {B}_{r}}{\partial z}|}_{0}-2\frac{{B}_{z0}}{d})}\end{array}$$

Similar considerations can be done for transducers placed along the axial direction, leading to a slightly different set of equations:

21 $$\begin{array}{rcl}{B}_{m}({H}_{13})-{B}_{m}({H}_{11}) & = & 2{\frac{\partial {B}_{z}}{\partial r}|}_{0}({y}_{O^{\prime} }-\frac{{v}_{2}}{2})+2\frac{{R}_{0}}{d}{\frac{\partial {B}_{z}}{\partial z}|}_{0}{v}_{2}+2\frac{{B}_{r0}}{d}{v}_{2}\\ {B}_{m}({H}_{14})-{B}_{m}({H}_{12}) & = & 2{\frac{\partial {B}_{z}}{\partial r}|}_{0}({x}_{O^{\prime} }-\frac{{v}_{1}}{2})+2\frac{{R}_{0}}{d}{\frac{\partial {B}_{z}}{\partial z}|}_{0}{v}_{1}+2\frac{{B}_{r0}}{d}{v}_{1}\\ {B}_{m}({H}_{23})-{B}_{m}({H}_{21}) & = & 2{\frac{\partial {B}_{z}}{\partial r}|}_{0}({y}_{O^{\prime} }+\frac{{v}_{2}}{2})-2\frac{{R}_{0}}{d}{\frac{\partial {B}_{z}}{\partial z}|}_{0}{v}_{2}-2\frac{{B}_{r0}}{d}{v}_{2}\\ {B}_{m}({H}_{24})-{B}_{m}({H}_{22}) & = & 2{\frac{\partial {B}_{z}}{\partial r}|}_{0}({x}_{O^{\prime} }+\frac{{v}_{1}}{2})-2\frac{{R}_{0}}{d}{\frac{\partial {B}_{z}}{\partial z}|}_{0}{v}_{1}-2\frac{{B}_{r0}}{d}{v}_{1}\end{array}$$

Thus, it can be easily derived that:

22 $$\begin{array}{rcl}{x}_{O^{\prime} } & = & \frac{[{B}_{m}({H}_{24})-{B}_{m}({H}_{22})]+[{B}_{m}({H}_{14})-{B}_{m}({H}_{12})]}{4{\frac{\partial {B}_{z}}{\partial r}|}_{0}}\\ {y}_{O^{\prime} } & = & \frac{[{B}_{m}({H}_{23})-{B}_{m}({H}_{21})]+[{B}_{m}({H}_{13})-{B}_{m}({H}_{11})]}{4{\frac{\partial {B}_{z}}{\partial r}|}_{0}}\\ {v}_{1} & = & \frac{[{B}_{m}({H}_{14})-{B}_{m}({H}_{12})]-[{B}_{m}({H}_{24})-{B}_{m}({H}_{22})]}{2(2\frac{{R}_{0}}{d}{\frac{\partial {B}_{z}}{\partial z}|}_{0}-{\frac{\partial {B}_{z}}{\partial r}|}_{0}-2\frac{{B}_{r0}}{d})}\\ {v}_{2} & = & \frac{[{B}_{m}({H}_{13})-{B}_{m}({H}_{11})]-[{B}_{m}({H}_{23})-{B}_{m}({H}_{21})]}{2(2\frac{{R}_{0}}{d}{\frac{\partial {B}_{z}}{\partial z}|}_{0}-{\frac{\partial {B}_{z}}{\partial r}|}_{0}-2\frac{{B}_{r0}}{d})}\end{array}$$

A further realization of the method can be obtained by only three transducers per solenoid side in order to minimize the system complexity. This is discussed in the appendix A. However, a more complex analytical model, and a higher uncertainty, both deriving from the lack of symmetry introduced removing one of the transducers per side, are involved.

## Proof of Principle and Method Optimization

The proposed method for monitoring the solenoid axis misalignment is summarized by (19) and (21) when employing radially- or axially-placed uniaxial Hall transducers, respectively. The measurement procedure is summed up as follows:

*At installation*:

- Place the transducers by defining *R*_0_ and *d*;
- Obtain the needed field components and derivatives at (*R*_0_, −*d*/2) and (*R*_0_, +*d*/2) in the solenoid reference system;
  *In operation*:
- Measure *B*_*m*_ at the Hall transducers points;
- Compute *x*_*O*′_, *y*_*O*′_, *v*_1_, and *v*_2_;
- Compute the related uncertainties.

The parameters defining the magnetic axis misalignment depend on the longitudinal and radial positions of the transducers (*R*_0_ and *d*), as well as on the specific longitudinal and radial field profile of the solenoid (represented by the field derivatives). Therefore, the transducers must be placed by taking into account that different positions can lead to a different uncertainty of the final result. Moreover, the misalignment parameters depend on the field profile, therefore, in general, an optimal placement of the transducers cannot be found. Instead, the optimization must be carried out by evaluating the uncertainty for the specific solenoid according its field.

This optimization procedure is highlighted by two case studies presented in this section, referring to two different models of a single coil of the multi-coil magnet to be employed in the ELI-NP project^25^. First, an analytical model for the single solenoid is considered, obtained on the basis of the geometries of a single coil of the ELI-NP magnet. Then, a FE model of the coil was obtained from the FEM of the multi-coil solenoid as a whole, by extracting the field of one of the coils.

Details about the employed field models are discussed in the next subsection. Then, the uncertainty is assessed and the optimal placement of the transducers is determined. Finally, simulations proving the effectiveness of the method in identifying the misalignment axis are reported.

### Solenoid field models

#### Analytical model

**Figure 6:**
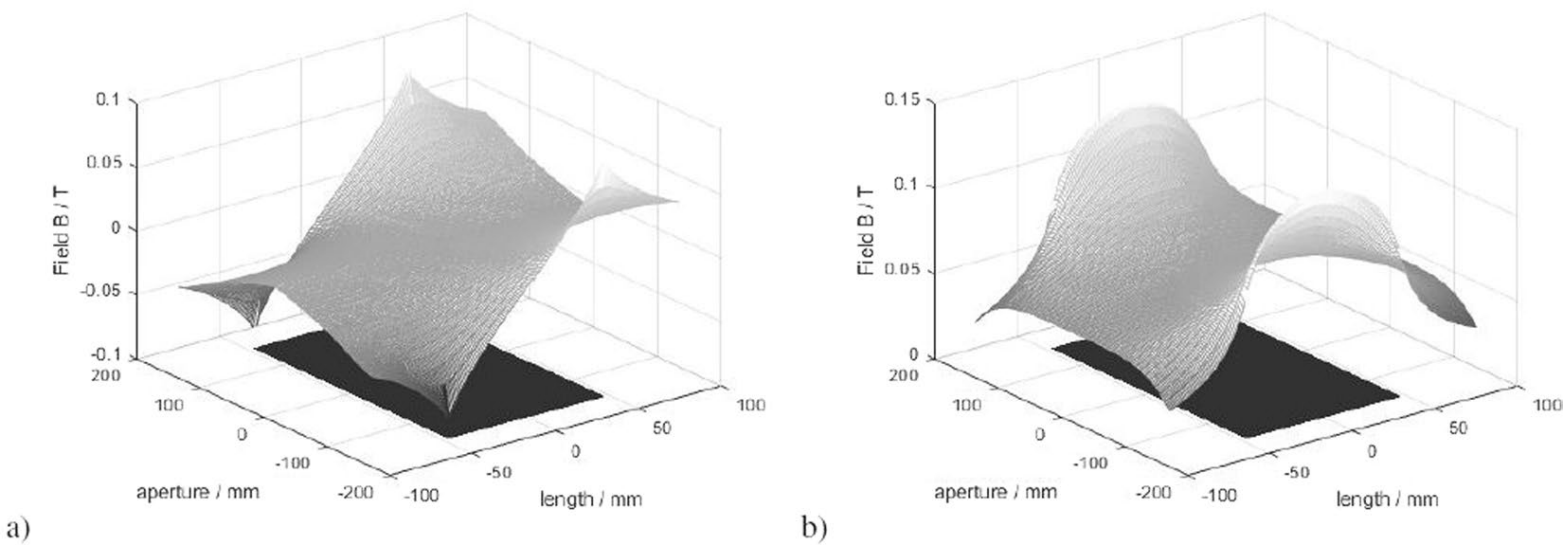
Radial (**a**) and axial (**b**) components of B for a multilayer solenoid at the plane *y* = 0 calculated with an analytical model based on a generalized elliptic integral.

A multi–solenoid made of 12 identical coils (the ELI-NP solenoid type B) was tested at the manufacturer (Danfysik) facility in 2015. In this paper, an analytical model of one of the coils of the magnet is considered. Among the models proposed in the scientific literature, a model of 2009 from MIT and the Community College of Vermont^26^ is taken into account. This model is based on a generalized elliptic integral that is easily computable with a fast numerical algorithm, and it allows to calculate the magnetic flux density of a single layer solenoid. In particular, the solenoid was modeled in MATLAB with geometrical parameters such as the number of turns (*N*), the length (*L*), the aperture radius (*R*), and the operating current (*I*_*op*_). The field of a multi-layer solenoid can be also simulated as a superposition of single layer solenoids with increasing aperture radius. Figure 6 shows *B*_*r*_ and *B*_*z*_ at the plane *y* = 0 for *x* ∈ [−*R*; +*R*] and *z* ∈ [−*L*; +*L*]. Due to the axisymmetry, the field for half a plane would be enough, but the entire plane is shown for clarity. The solenoid model has 12 layers, 9 turns per layer, length equal to 93 mm, aperture radius equal to 153,1 mm, and operating current *I*_*op*_ = 177 A. Such parameters were chosen according to the characteristics of a single coil of the ELI-NP type B solenoid. In the figure, the section at *y* = 0 of the solenoid is also shown as a rectangle under the surface representing the field components, to better highlight the solenoid dimensions.

#### Finite Element Model

During the factory acceptance test (F.A.T.) of the ELI-NP multi-solenoid Type B, several magnetic, electrical, and hydraulic measurements have been performed, including a magnetic flux density mapping along several paths for two configurations: (i) by powering all the coils with the same current polarity (+ + + + configuration), and (ii) by alternating the current polarity for each coil triplet (+ + − − configuration). However, for both the configurations, only the longitudinal component of the flux density (*B*_*z*_) was measured, so such measurements were not used directly to assess the derivatives needed for the measurement methods, because also the radial flux density would be required. Conversely, a FEM model was exploited and the longitudinal component output from the FEM was compared with the measured data.

A finite element analysis (F.E.A.) of the ELI-NP multi-solenoid type B was performed using Poisson Superfish software, in order to map the flux density generated by the twelve solenoid coils operating in DC. The simulation is a 2D model of the magnet with cylindrical symmetry, where all the coils and iron yoke dimensions were obtained by mechanical drawings and dimensional tests supplied by Danfysik. Each coil, composed by 108 turns, was powered with the nominal current of 177 A reaching an Ampere·Turns value of 19.116 A. The iron yoke material was modeled with a 17-4 ph stainless steel first magnetization curve. It is a mild steel, approximating the original iron yoke material (steel 37) satisfyingly. The output of the simulation is a field map of the radial and axial flux density components *B*_*r*_ and *B*_*z*_, assessed in the inner aperture coil air region, with a corresponding step of 2 mm and 1 mm, respectively. Also all the derivatives of *B*_*z*_ and *B*_*r*_ in radial and longitudinal direction were included in the output files.

**Figure 7:**
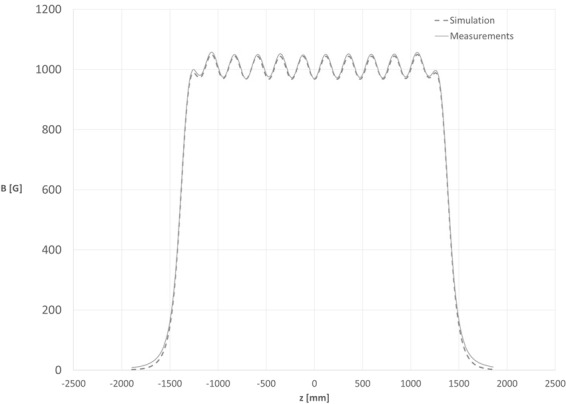
Simulated and Measured Longitudinal Component of the Magnetic Field along the magnet axis.

The measured *B*_*z*_ component measured during the F.A.T. was compared with the *B*_*z*_ calculated through the simulations. As shown in Fig. 7, excluding the ends part of the fringe fields, the curves matched significantly within 2%. This error is negligible with respect to the measured and the simulated total integral field, over several trajectories at different radius from the mechanical axis (Table 1).

**Table 1:** Comparison between Simulated and Measured Field Integral.

	Simulated Field integral [T·mm]	Measured Field integral [T·mm]	Δ Field Integral [%]
*R* = 0 mm	287.7348	284.3833	1.2
*R* = 30 mm	287.7259	284.3843	1.2
*R* = 60 mm	287.7341	284.3871	1.2
*R* = 90 mm	287.8302	284.3914	1.2
*R* = 120 mm	287.9348	284.4397	1.2

**Figure 8:**
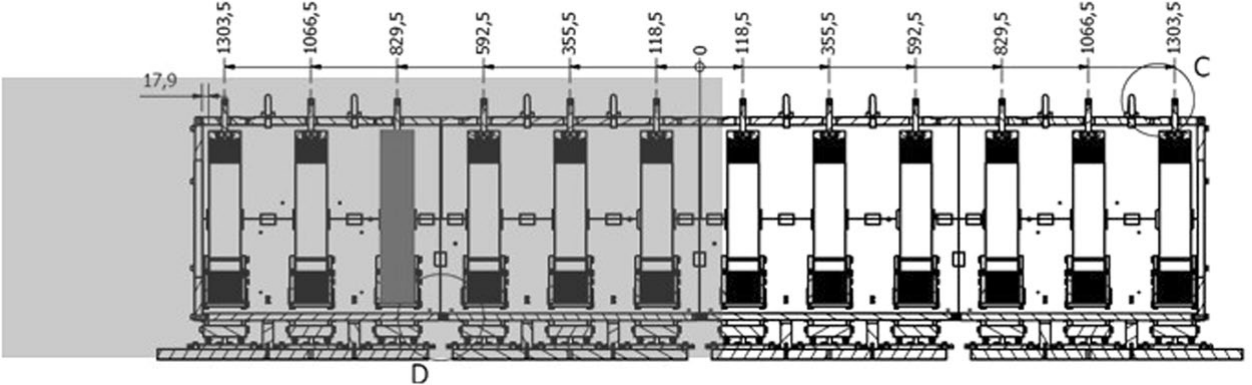
Geometry of the ELI-NP multi-coil magnet. The coil supplied for the single solenoid model simulation is highlighted (gray) together with the spatial region of interest for the FEM (light gray).

By relying on the same magnet model, only one coil per time can be supplied in order to obtain the field irradiated by a single solenoid. In Fig. 8, the coil considered for the FEM model is highlighted. This is the third of the first triplet and the field was simulated in the spatial region also highlighted in a lighter gray. The results of this simulation were employed for optimizing the transducers placement and validating the method.

### Uncertainty Assessment

The uncertainties of the *O*′ coordinates, *v*_1_, and *v*_2_ were assessed by considering their explicit expressions, obtained by exploiting the symmetries of the magnetic flux density irradiated by the solenoid. In particular, for radial transducers, the (20) and (22) have to be considered, for radial and axial Hall transducers, respectively. The uncertainty was computed by using the “law of propagation of the uncertainties” (LPU) for each equation. For the sake of brevity, only *x*_*O*′_ and *v*_1_ for radial transducers are here considered, but the same reasoning can be applied for *y*_*o*′_ and *v*_2_, and for axial transducers. The squared standard uncertainties are:

23 $$\begin{array}{rcl}{u}_{{x}_{O^{\prime} }}^{2} & = & \frac{{u}_{B}^{2}}{4{({\frac{\partial {B}_{r}}{\partial r}|}_{0})}^{2}}+{(\frac{{x}_{o^{\prime} }}{{\frac{\partial {B}_{r}}{\partial r}|}_{0}})}^{2}{u}_{\partial }^{2}\\ {u}_{{v}_{1}}^{2} & = & \frac{{u}_{B}^{2}}{{({\frac{\partial {B}_{r}}{\partial r}|}_{0}-2\frac{{R}_{0}}{d}{\frac{\partial {B}_{r}}{\partial z}|}_{0}-2\frac{{B}_{z0}}{d})}^{2}}+{(\frac{{v}_{1}}{{\frac{\partial {B}_{r}}{\partial r}|}_{0}-2\frac{{R}_{0}}{d}{\frac{\partial {B}_{r}}{\partial z}|}_{0}-2\frac{{B}_{z0}}{d}})}^{2}\\  &  & \times \,[(1+4\frac{{R}_{0}^{2}}{{d}^{2}}){u}_{\partial }^{2}+\frac{4}{{d}^{2}}{u}_{f}^{2}]+{(\frac{{v}_{1}}{{\frac{\partial {B}_{r}}{\partial r}|}_{0}-2\frac{{R}_{0}}{d}{\frac{\partial {B}_{r}}{\partial z}|}_{0}-2\frac{{B}_{z0}}{d}})}^{2}\\  &  & \times \,[4{(\frac{{B}_{z0}}{{d}^{2}}+\frac{{R}_{0}}{{d}^{2}}{\frac{\partial {B}_{r}}{\partial z}|}_{0})}^{2}+\frac{4}{{d}^{2}}{\frac{\partial {B}_{r}}{\partial z}|}_{0}^{2}]{u}_{g}^{2}\end{array}$$

where, *u*_*B*_ is the uncertainty of the values measured by the transducers, supposed equal for all the transducers outputs; *u*_*g*_ is the uncertainty related to the transducers placing, supposed equal for both geometrical parameters, *R*_0_ and *d*; *u*_∂_ is the uncertainty of the derivatives of *B*_*r*_, and *u*_*f*_ is the uncertainty of the *B*_*z*0_ value. The last contributions arise from the field model, and they express the goodness of fitting with respect to the measured field. Once the above uncertainties are known, ${u}_{{x}_{O^{\prime} }}$ and ${u}_{{v}_{1}}$ are expressed as a function of (*R*_0_, *d*) in order to find the optimal placement.

Assuming typical values of *u*_*B*_ ≈ 10^−4^ T for Hall transducers and *u*_*R*0_ ≈ *u*_*d*_ = *u*_*g*_ ≈ 10^−5^ m, in a placement by laser tracking. Moreover, considerations about the field model suggest that derivatives and field components uncertainties are in the order of percent of their respective values.

To have a better understanding of the contributions to the total uncertainty, it is possible to demonstrate that the uncertainty related to the placement parameters is negligible with respect to that of the field measurements, under the above assumptions. This can be understood by noticing that:

24 $$\begin{array}{l}(1+4\frac{{R}_{0}^{2}}{{d}^{2}}){u}_{\partial }^{2}+\frac{4}{{d}^{2}}{u}_{f}^{2}\gg 4{(\frac{{B}_{z0}}{{d}^{2}}+\frac{{R}_{0}}{{d}^{2}}{\frac{\partial {B}_{r}}{\partial z}|}_{0})}^{2}{u}_{g}^{2}+\frac{4}{{d}^{2}}{\frac{\partial {B}_{r}}{\partial z}|}_{0}^{2}{u}_{g}^{2}.\end{array}$$

According to the adopted field values, in fact, the derivatives values are of the order of 1 T/m, *B*_*z*0_ is about 0.06 T in the points of interest, and *u*_*g*_ ≈ 10^−5^ m. For the considered solenoid, therefore, the second term is in the order of 10^−4^ m. Given the values of the derivatives and *B*_*z*0_, *u*_∂_ ≈ 10^−2^ T/m, while *u*_*f*_ ≈ 10^−3^ T, when the relative uncertainties arising from the field model are within the order of percent. Moreover, the contribution related to *u*_∂_ is dominant at the first member, because *u*_*f*_ is an order of magnitude below, while their coefficients are of the same order. Finally, the coordinates of *O*′ and the values of *v*_1_ (and *v*_2_) are below 1 mm for a small misalignment. Thus, considering only the uncertainty contributions of *u*_*B*_ and *u*_∂_, (23) can be rewritten as:

25 $$\begin{array}{rcl}{u}_{xo^{\prime} }^{2} & \approx  & \frac{{u}_{B}^{2}}{4{({\frac{\partial {B}_{r}}{\partial r}|}_{0})}^{2}}\\ {u}_{v1}^{2} & \approx  & \frac{{u}_{B}^{2}+{v}_{1}^{2}(1+4\frac{{R}_{0}^{2}}{{d}^{2}}){u}_{\partial }^{2}}{{({\frac{\partial {B}_{r}}{\partial r}|}_{0}-2\frac{{R}_{0}}{d}{\frac{\partial {B}_{r}}{\partial z}|}_{0}-2\frac{{B}_{z0}}{d})}^{2}}\end{array}$$

The uncertainty minimization is hence limited by the Hall transducers technology, even if this can be mitigated by the transducers placement (minimization of the denominators).

As an example, when monitoring the solenoid magnetic axis, there could be interest in the point $({x}_{{O}_{2}^{^{\prime} }},{y}_{{O}_{2}^{^{\prime} }},{z}_{{O}_{2}^{^{\prime} }})=$ $({x}_{O^{\prime} }+{v}_{1}/2,{y}_{O^{\prime} }+{v}_{2}/2,+\,d/2)$, and one of the uncertainties of interest would be:

26 $${u}_{{x}_{{O}_{2}^{^{\prime} }}}=\sqrt{{u}_{{x}_{O^{\prime} }}^{2}+\frac{{u}_{v1}^{2}}{4}}$$

**Figure 9:**
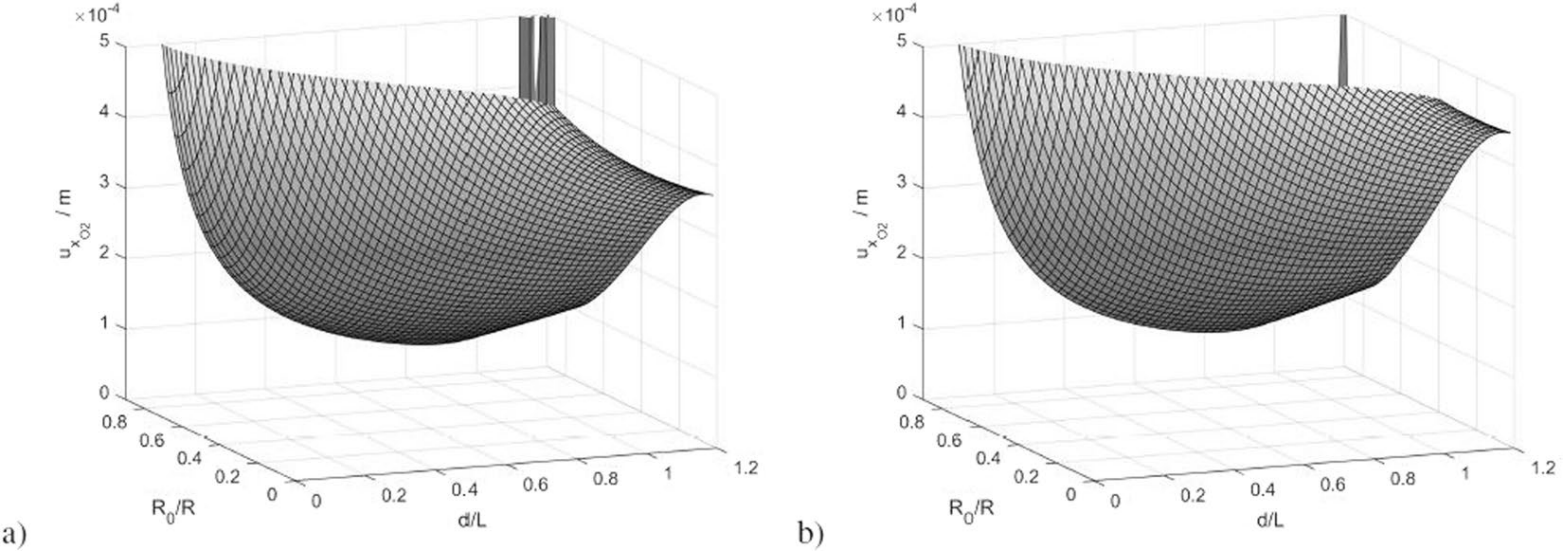
Uncertainty of the “x” coordinate of ${O^{\prime} }_{2}$ for the case of radially placed Hall transducers, calculated with the analytical model (**a**) and with the FEM model (**b**).

The values of this uncertainty as a function of (*R*_0_, *d*) are shown in Fig. 9, when employing the analytical model or the FEM. The minimum is obtained when *R*_0_ is the 90% of the aperture, and *d* equals about the 90% of the solenoid length for both cases, thus the transducers have to be placed slightly inside the magnet aperture to minimize ${u}_{{x}_{{O}_{2}^{^{\prime} }}}$. Similar considerations stand for ${u}_{{y}_{{O}_{2}^{^{\prime} }}}$ and the optimal placement could aim to minimize both the uncertainties separately, or their combination.

Additional considerations are needed for the three transducers case, as reported in the appendix A.

### Method validation through simulations

Once fixed *R*_0_ and *d*, the effectiveness of the proposed method can be proved by simulation, through either the analytical or FE field model. A known misalignment is imposed in order to calculate the reference quantities identifying the magnetic axis (reference values of *O*′, *v*_1_, and *v*_2_). Then, these quantities are estimated by the equations of the method, and a corresponding uncertainty can be assessed. In the simulation, the misalignment is implemented with a rotation and then a translation of the solenoid. When employing the analytical field model, simulation results show that the estimated error is always below 10^−7^ m. when the misaligned magnetic axis is rotated of angles up to *π*/1000 and translated of 1/1000 times the solenoid length. These are reasonable maximum misalignments in the cases of interest. Taking into account the same misalignment, the uncertainty is higher but always below 10^−6^ m, when employing the FE model.

## Conclusions

In this work, a novel method for monitoring in real time the magnetic axis misalignment in solenoids has been presented. Relying on few measurements of the magnetic flux density, this method is especially intended for applications where the magnet aperture is not accessible. The magnetic axis is determined calculating the solenoid center and slope parameters. To this aim, a field model of the solenoid must also be employed to extract useful data for the calculations.

The mathematical model of the method was derived for the case of radial or axial placement of the Hall transducers, and for the case of four or three transducers per solenoid side.

A case study, based on the ELI-NP type B multi-coil solenoid, was considered. In particular, an analytical and a FE model of a single coil of the ELI-NP type B solenoid were employed to assess the uncertainty of the method and to obtain the optimal placement of the Hall transducers. Moreover, simulations were carried out with both the models, to verify the capability of the method of estimating an imposed misalignment. Further work will be directed toward an experimental evaluation of the method on a actual solenoid, as well as to its extension to the case of a multi–coil solenoid.

## A Three Transducers Per Solenoid Side

**Figure 10:**
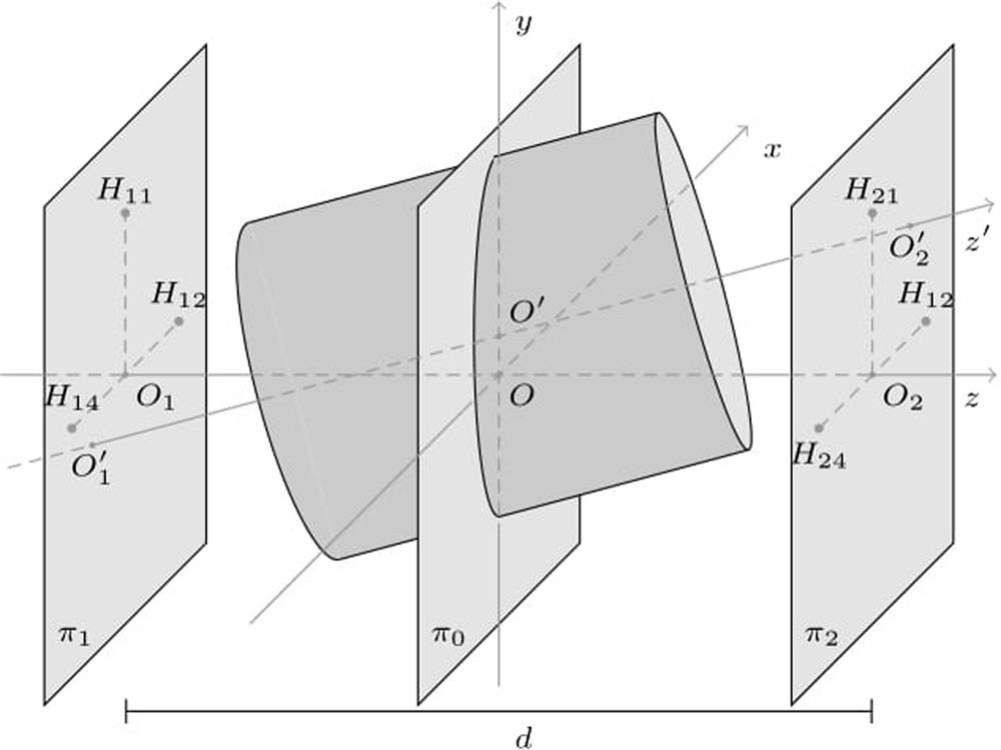
Monitoring the magnetic axis misalignments with three Hall transducers per solenoid side.

Adopting only three Hall transducers per solenoid side, as shown in Fig. 10, it is still possible to obtain a system of equation to calculate *O*′, *v*_1_, and *v*_2_. The position of the transducers are:

$$\begin{array}{cc}{H}_{11}=(0,+\,{R}_{0},-\,d/2) & {H}_{21}=(0,+\,{R}_{0},d/2)\\ {H}_{12}=(\,+\,{R}_{0},0,-\,d/2) & {H}_{22}=(\,+\,{R}_{0},0,d/2)\\ {H}_{14}=(\,-\,{R}_{0},0,-\,d/2) & {H}_{24}=(\,-\,{R}_{0},0,d/2)\end{array}$$

In this case, the couples are (*H*_14_, *H*_12_), (*H*_24_, *H*_22_), (*H*_14_, *H*_11_), and (*H*_24_, *H*_21_). For radial transducers, the equations of interest are:

27 $$\begin{array}{rcl}{B}_{m}({H}_{14})-{B}_{m}({H}_{12}) & = & -2{\frac{\partial {B}_{r}}{\partial r}|}_{0}({x}_{O^{\prime} }-\frac{{v}_{1}}{2})-2\frac{{R}_{0}}{d}{\frac{\partial {B}_{r}}{\partial z}|}_{0}{v}_{1}-2\frac{{v}_{1}}{d}{B}_{z0}\\ {B}_{m}({H}_{24})-{B}_{m}({H}_{22}) & = & +2{\frac{\partial {B}_{r}}{\partial r}|}_{0}({x}_{O^{\prime} }+\frac{{v}_{1}}{2})-2\frac{{R}_{0}}{d}{\frac{\partial {B}_{r}}{\partial z}|}_{0}{v}_{1}-2\frac{{v}_{1}}{d}{B}_{z0}\\ {B}_{m}({H}_{14})-{B}_{m}({H}_{11}) & = & -{\frac{\partial {B}_{r}}{\partial r}|}_{0}[{x}_{O^{\prime} }-\frac{{v}_{1}}{2}+{y}_{O^{\prime} }-\frac{{v}_{2}}{2}+\frac{{R}_{0}}{{d}^{2}}({v}_{2}^{2}-{v}_{1}^{2})]\\  &  & -\,(\frac{{R}_{0}}{d}{\frac{\partial {B}_{r}}{\partial z}|}_{0}+\frac{{B}_{z0}}{d})({v}_{1}+{v}_{2})\\ {B}_{m}({H}_{24})-{B}_{m}({H}_{21}) & = & +{\frac{\partial {B}_{r}}{\partial r}|}_{0}[{x}_{O^{\prime} }+\frac{{v}_{1}}{2}+{y}_{O^{\prime} }+\frac{{v}_{2}}{2}+\frac{{R}_{0}}{{d}^{2}}({v}_{2}^{2}-{v}_{1}^{2})]\\  &  & -\,(\frac{{R}_{0}}{d}{\frac{\partial {B}_{r}}{\partial z}|}_{0}+\frac{{B}_{z0}}{d})({v}_{1}+{v}_{2})\end{array}$$

These equations can be better re-arranged neglecting the quadratic terms of *v*_1_ and *v*_2_, and defining:

28 $$\begin{array}{rcl}{K}_{1} & = & {B}_{m}({H}_{14})-{B}_{m}({H}_{11})+{\frac{\partial {B}_{r}}{\partial r}|}_{0}({x}_{O^{\prime} }-\frac{{v}_{1}}{2})+(\frac{{R}_{0}}{d}{\frac{\partial {B}_{r}}{\partial z}|}_{0}+\frac{{B}_{z0}}{d}){v}_{1}\\ {K}_{2} & = & {B}_{m}({H}_{24})-{B}_{m}({H}_{21})-{\frac{\partial {B}_{r}}{\partial r}|}_{0}({x}_{O^{\prime} }+\frac{{v}_{1}}{2})+(\frac{{R}_{0}}{d}{\frac{\partial {B}_{r}}{\partial z}|}_{0}+\frac{{B}_{z0}}{d}){v}_{1}\end{array}$$

This also means that the first two equations have to be solved before in order to obtain *x*_*O*′_ and *v*_1_, which are needed to calculate *K*_1_ and *K*_2_. Thanks to that, (29) hold:

29 $$\begin{array}{rcl}{B}_{m}({H}_{14})-{B}_{m}({H}_{12}) & = & -2{\frac{\partial {B}_{r}}{\partial r}|}_{0}({x}_{O^{\prime} }-\frac{{v}_{1}}{2})-2\frac{{R}_{0}}{d}{\frac{\partial {B}_{r}}{\partial z}|}_{0}{v}_{1}-2\frac{{v}_{1}}{d}{B}_{z0}\\ {B}_{m}({H}_{24})-{B}_{m}({H}_{22}) & = & +2{\frac{\partial {B}_{r}}{\partial r}|}_{0}({x}_{O^{\prime} }+\frac{{v}_{1}}{2})-2\frac{{R}_{0}}{d}{\frac{\partial {B}_{r}}{\partial z}|}_{0}{v}_{1}-2\frac{{v}_{1}}{d}{B}_{z0}\\ {K}_{1} & = & -{\frac{\partial {B}_{r}}{\partial r}|}_{0}({y}_{O^{\prime} }-\frac{{v}_{2}}{2})-(\frac{{R}_{0}}{d}{\frac{\partial {B}_{r}}{\partial z}|}_{0}+\frac{{B}_{z0}}{d}){v}_{2}\\ {K}_{2} & = & +{\frac{\partial {B}_{r}}{\partial r}|}_{0}({y}_{O^{\prime} }+\frac{{v}_{2}}{2})-(\frac{{R}_{0}}{d}{\frac{\partial {B}_{r}}{\partial z}|}_{0}+\frac{{B}_{z0}}{d}){v}_{2}\end{array}$$

Eq. (29) allow to obtain more explicit expressions for *x*_*O*′_, *y*_*O*′_, *v*_1_, and *v*_2_:

30 $$\begin{array}{rcl}{x}_{o^{\prime} } & = & \frac{[{B}_{m}({H}_{24})-{B}_{m}({H}_{22})]-[{B}_{m}({H}_{14})-{B}_{m}({H}_{12})]}{4{\frac{\partial {B}_{r}}{\partial r}|}_{0}}\\ {y}_{o^{\prime} } & = & \frac{{K}_{2}-{K}_{1}}{2{\frac{\partial {B}_{r}}{\partial r}|}_{0}}\\ {v}_{1} & = & \frac{[{B}_{m}({H}_{24})-{B}_{m}({H}_{22})]+[{B}_{m}({H}_{14})-{B}_{m}({H}_{12})]}{2({\frac{\partial {B}_{r}}{\partial r}|}_{0}-2\frac{{R}_{0}}{d}{\frac{\partial {B}_{r}}{\partial z}|}_{0}-2\frac{{B}_{z0}}{d})}\\ {v}_{2} & = & \frac{{K}_{2}+{K}_{1}}{{\frac{\partial {B}_{r}}{\partial r}|}_{0}-2\frac{{R}_{0}}{d}{\frac{\partial {B}_{r}}{\partial z}|}_{0}-2\frac{{B}_{z0}}{d}}\end{array}$$

Analogous equations hold for axial transducers:

31 $$\begin{array}{rcl}{B}_{m}({H}_{14})-{B}_{m}({H}_{12}) & = & 2{\frac{\partial {B}_{z}}{\partial r}|}_{0}({x}_{O^{\prime} }-\frac{{v}_{1}}{2})+2\frac{{R}_{0}}{d}{\frac{\partial {B}_{z}}{\partial z}|}_{0}{v}_{1}+2\frac{{B}_{r0}}{d}{v}_{1}\\ {B}_{m}({H}_{24})-{B}_{m}({H}_{22}) & = & 2{\frac{\partial {B}_{z}}{\partial r}|}_{0}({x}_{O^{\prime} }+\frac{{v}_{1}}{2})-2\frac{{R}_{0}}{d}{\frac{\partial {B}_{z}}{\partial z}|}_{0}{v}_{1}-2\frac{{B}_{r0}}{d}{v}_{1}\\ {K}_{1} & = & {\frac{\partial {B}_{z}}{\partial r}|}_{0}({y}_{O^{\prime} }-\frac{{v}_{2}}{2})+(\frac{{R}_{0}}{d}{\frac{\partial {B}_{z}}{\partial z}|}_{0}-\frac{{B}_{r0}}{d}){v}_{2}\\ {K}_{2} & = & {\frac{\partial {B}_{z}}{\partial r}|}_{0}({y}_{O^{\prime} }+\frac{{v}_{2}}{2})-(\frac{{R}_{0}}{d}{\frac{\partial {B}_{z}}{\partial z}|}_{0}-\frac{{B}_{r0}}{d}){v}_{2}\end{array}$$

obtained defining:

32 $$\begin{array}{rcl}{K}_{1} & = & {B}_{m}({H}_{14})-{B}_{m}({H}_{11})-{\frac{\partial {B}_{z}}{\partial r}|}_{0}({x}_{O^{\prime} }-\frac{{v}_{1}}{2})-(\frac{{R}_{0}}{d}{\frac{\partial {B}_{z}}{\partial z}|}_{0}-\frac{{B}_{r0}}{d}){v}_{1}\\ {K}_{2} & = & {B}_{m}({H}_{24})-{B}_{m}({H}_{21})-{\frac{\partial {B}_{z}}{\partial r}|}_{0}({x}_{O^{\prime} }+\frac{{v}_{1}}{2})+(\frac{{R}_{0}}{d}{\frac{\partial {B}_{z}}{\partial z}|}_{0}-\frac{{B}_{r0}}{d}){v}_{1}\end{array}$$

The expressions of interest in this case are:

33 $$\begin{array}{rcl}{x}_{o^{\prime} } & = & \frac{[{B}_{m}({H}_{24})-{B}_{m}({H}_{22})]+[{B}_{m}({H}_{14})-{B}_{m}({H}_{12})]}{4{\frac{\partial {B}_{z}}{\partial r}|}_{0}}\\ {y}_{o^{\prime} } & = & \frac{{K}_{2}+{K}_{1}}{2{\frac{\partial {B}_{z}}{\partial r}|}_{0}}\\ {v}_{1} & = & \frac{[{B}_{m}({H}_{14})-{B}_{m}({H}_{12})]-[{B}_{m}({H}_{24})-{B}_{m}({H}_{22})]}{2(2\frac{{R}_{0}}{d}{\frac{\partial {B}_{z}}{\partial z}|}_{0}-{\frac{\partial {B}_{z}}{\partial r}|}_{0}-2\frac{{B}_{r0}}{d})}\\ {v}_{2} & = & \frac{{K}_{2}-{K}_{1}}{{\frac{\partial {B}_{z}}{\partial r}|}_{0}-2\frac{{R}_{0}}{d}{\frac{\partial {B}_{z}}{\partial z}|}_{0}+2\frac{{B}_{r0}}{d}}\end{array}$$

Regarding the uncertainties, while for *x*_*O*′_ and *v*_1_ the expressions are the same of the four transducers case, for *y*_*O*′_ and *v*_2_ the expressions are different. Applying the LPU to (30), which are valid for radially placed transducers, eq. (34) hold:

34 $$\begin{array}{rcl}{u}_{yo^{\prime} }^{2} & = & \frac{{u}_{{K}_{2}-{K}_{1}}^{2}}{{(2{\frac{\partial {B}_{r}}{\partial r}|}_{0})}^{2}}+{(\frac{{y}_{O^{\prime} }}{{\frac{\partial {B}_{r}}{\partial r}|}_{0}})}^{2}{u}_{\partial }^{2}\\ {u}_{v2}^{2} & = & \frac{{u}_{{K}_{2}+{K}_{1}}^{2}}{{({\frac{\partial {B}_{r}}{\partial r}|}_{0}-2\frac{{R}_{0}}{d}{\frac{\partial {B}_{r}}{\partial z}|}_{0}-2\frac{{B}_{z0}}{d})}^{2}}+{(\frac{{v}_{2}}{{\frac{\partial {B}_{r}}{\partial r}|}_{0}-2\frac{{R}_{0}}{d}{\frac{\partial {B}_{r}}{\partial z}|}_{0}-2\frac{{B}_{z0}}{d}})}^{2}\\  &  & \times \,[(1+\frac{4{R}_{0}^{2}}{{d}^{2}}){u}_{\partial }^{2}+\frac{4}{{d}^{2}}{u}_{f}^{2}]+{(\frac{{v}_{2}}{{\frac{\partial {B}_{r}}{\partial r}|}_{0}-2\frac{{R}_{0}}{d}{\frac{\partial {B}_{r}}{\partial z}|}_{0}-2\frac{{B}_{z0}}{d}})}^{2}\\  &  & \times \,[4{(\frac{{R}_{0}}{{d}^{2}}{\frac{\partial {B}_{r}}{\partial z}|}_{0}+\frac{{B}_{z0}}{{d}^{2}})}^{2}+\frac{4}{{d}^{2}}{\frac{\partial {B}_{r}}{\partial z}|}_{0}^{2}]{u}_{g}^{2}\end{array}$$

In these expressions, ${u}_{K2-K1}^{2}$ and ${u}_{K2+K1}^{2}$ have to be computed by applying the LPU to the expression of *K*_2_ − *K*_1_ and *K*_2_ + *K*_1_, which can be obtained through (28). The following equations hold:

35 $$\begin{array}{rcl}{u}_{{K}_{2}-{K}_{1}}^{2} & = & 4{u}_{B}^{2}+4{\frac{\partial {B}_{r}}{\partial r}|}_{0}^{2}{u}_{xo^{\prime} }^{2}+4{x}_{O^{\prime} }^{2}{u}_{\partial }^{2}\\ {u}_{{K}_{2}+{K}_{1}}^{2} & = & \frac{17}{2}{u}_{B}^{2}\end{array}$$

In (35), the contribution from *u*_∂_ in the first equation is negligible with respect to the other terms noticing that the coefficient of this term is small, and remembering that *u*_∂_ ≈ 10^−2^ T/m. Recalling eq. (25), in conclusion it is ${u}_{{K}_{2}-{K}_{1}}^{2}\approx 5{u}_{B}^{2}$. Then, eq. (34) can be approximated as:

36 $$\begin{array}{rcl}{u}_{yo^{\prime} }^{2} & \approx  & \frac{5{u}_{B}^{2}}{4{({\frac{\partial {B}_{r}}{\partial r}|}_{0})}^{2}}\\ {u}_{v2}^{2} & = & \frac{8.5{u}_{B}^{2}+{v}_{2}^{2}(1+\frac{4{R}_{0}^{2}}{{d}^{2}}){u}_{\partial }^{2}}{{({\frac{\partial {B}_{r}}{\partial r}|}_{0}-2\frac{{R}_{0}}{d}{\frac{\partial {B}_{r}}{\partial z}|}_{0}-2\frac{{B}_{z0}}{d})}^{2}}\end{array}$$

**Figure 11:**
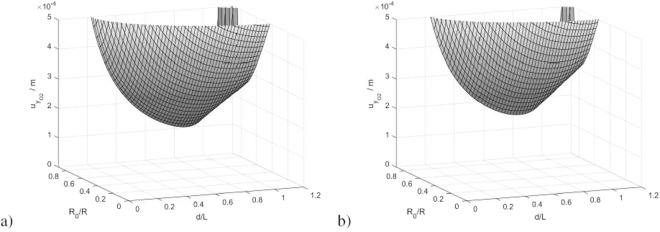
Uncertainty of the “y” coordinate of ${O}_{2}^{^{\prime} }$ for the case of three radial Hall transducers per solenoid side, calculated with the analytical model (**a**) and with the FEM model (**b**).

In conclusion, owing to the lack of symmetry in the transducer placement, the uncertainties of *y*_*O*′_ and *v*_2_ are slightly higher than those of *x*_*O*′_ and *v*_1_. This difference can be seen referring to the uncertainty of ${y}_{{O}_{2}^{^{\prime} }}$, which is shown in Fig. 11. Instead, the uncertainty of ${x}_{{O}_{2}^{^{\prime} }}$ is still the one of Fig. 9.

The figure highlights that the uncertainty of ${y}_{{O}_{2}^{^{\prime} }}$ is higher than the one of ${x}_{{O}_{2}^{^{\prime} }}$, but the optimal placement is almost the same as the previous case, namely *R*_0_ equal about the 90% of the aperture and *d* equal about the 90% of the solenoid length.
